# Growing Up Through a Pandemic: A Mixed‐Methods Study of How the COVID‐19 Pandemic Shaped the Transition to Adulthood for Youth With Special Healthcare Needs and Their Families

**DOI:** 10.1111/cch.70294

**Published:** 2026-05-10

**Authors:** Lin Li, Jan Willem Gorter, Karen Cook, Alene Toulany, Paula Robeson, Jessica Geboers, Dilshad Kassam‐Lallani, Danijela Grahovac, Aline Bogossian, Rocio Gutierrez Rojas, Barb Galuppi, Dayle Mccauley, Anne Fournier

**Affiliations:** ^1^ Lawrence Bloomberg Faculty of Nursing University of Toronto Toronto Ontario Canada; ^2^ Centre for Addiction and Mental Health Toronto Ontario Canada; ^3^ University Medical Center Utrecht Utrecht the Netherlands; ^4^ Department of Pediatrics & CanChild Centre for Childhood‐onset Disability Research McMaster University Hamilton Ontario Canada; ^5^ Athabasca University Athabasca Alberta Canada; ^6^ Division of Adolescent Medicine The Hospital for Sick Children (SickKids) Toronto Ontario Canada; ^7^ Department of Paediatrics University of Toronto Toronto Ontario Canada; ^8^ SickKids Research Institute Toronto Ontario Canada; ^9^ Institute for Clinical Evaluative Sciences (ICES) Toronto Ontario Canada; ^10^ Children's Healthcare Canada Ottawa Ontario Canada; ^11^ CanChild Centre for Childhood‐onset Disability Research McMaster University Hamilton Ontario Canada; ^12^ Holland‐Bloorview Kids Rehabilitation Hospital Toronto Ontario Canada; ^13^ École de Travail Social Université de Montréal Montréal Quebec Canada; ^14^ Centre de Recherche Interdisciplinaire en Réadaptation de Montréal (CRIR) Montréal Quebec Canada; ^15^ CHU Sainte‐Justine Montréal Quebec Canada; ^16^ Cardiology Division CHU Mere‐Enfant Ste‐Justine Montréal Canada

**Keywords:** COVID‐19, emerging adulthood, health equity, mixed methods, patient‐oriented research, transition to adulthood, youth with special healthcare needs

## Abstract

**Background:**

The COVID‐19 pandemic profoundly altered daily life and interrupted key milestones during the transition to adulthood for youth with special healthcare needs (YSHCN). In this patient‐oriented study, we investigated the impact of the COVID‐19 pandemic on Canadian YSHCN during their transition to adulthood.

**Methods:**

We used a sequential, exploratory mixed methods design. In Phase 1 (Interpretive Description), we conducted semi‐structured interviews with 21 YSHCN or their caregiver proxies. Rapid qualitative analysis identified key themes that informed Phase 2, a cross‐sectional online survey completed by 516 YSHCN or their proxies. Survey results were summarized using descriptive statistics, and heterogeneity in experiences was examined using chi‐square and multivariable logistic regression analyses. Mixed methods findings were integrated using joint displays.

**Results:**

YSHCN described positive and negative experiences across the domains of healthcare, autonomy, relationships, vocation and lifestyle. Virtual modalities enhanced accessibility for some but deepened exclusion for others, particularly youth requiring hands‐on or specialized support. Participants described strengthened family connections and accelerated pathways to autonomy as positive experiences, whereas disrupted access to healthcare, delayed milestones and increased social isolation were common negative experiences. In the survey, family support and positive coping strategies emerged as protective influences, whereas lack of in‐person services, distressing news and social distancing were identified as key negative factors. Youth with mental or developmental healthcare needs were more likely to experience positive impacts of virtual services, whereas younger youth and those with mental healthcare needs were more likely to be negatively impacted by the lack of in‐person services.

**Conclusions:**

The pandemic had mixed impacts on YSHCN, highlighting the need for flexible health, education and community services that accommodate diverse needs. Findings underscore the importance of building inclusive, resilient systems and policies that preserve accessibility gains and meaningfully engage youth and caregivers in post‐pandemic recovery and future crisis planning.

## Summary

- The COVID‐19 pandemic disrupted access to health, education and community services for YSHCN.
- Virtual care improved accessibility for some but deepened exclusion for others, underscoring the need for flexible, individualized approaches in health services.
- Lack of in‐person services was the greatest contributor to negative experiences, with younger youth and those with mental healthcare needs more likely to be impacted.
- Family support and adaptive coping were key protective factors, highlighting the resilience of YSHCN and their families.
- Clinicians and policymakers must build adaptable systems and policies that safeguard YSHCN and other vulnerable populations during future crises.

## 1 Introduction

The COVID‐19 pandemic profoundly disrupted daily life, particularly for youth navigating the transition to adulthood. Public health measures such as school closures, restrictions on social gatherings and the widespread shift to virtual learning and work interrupted key developmental tasks of emerging adulthood, including the pursuit of independence, educational attainment, employment opportunities and relationship formation (Smyth and Nolan 2022). Numerous studies have reported adverse health and social outcomes for youth during the pandemic, including a rise in mental health problems, difficulties with remote learning, increased job precarity compared with adults in older age groups and amplified social isolation (Juvonen et al. 2022; Repo et al. 2023; Samji et al. 2022; Smyth and Nolan 2022). A series of lifestyle shifts also raised concerns during this period, including increases in unhealthy eating habits, screen time, and smoking or vaping, and decreased involvement in sports or other enjoyable activities (Dvorsky et al. 2023; Mazzolani et al. 2021; Sharma et al. 2024; Smyth and Nolan 2022).

Although the pandemic affected all youth broadly, it disproportionately impacted those with pre‐existing health or social vulnerabilities, such as those with chronic health conditions or disabilities, or those marginalized by race or socioeconomic status (Dvorsky et al. 2023; Nicholas et al. 2022; Schormans et al. 2021; Smyth and Nolan 2022). For youth with special healthcare needs (YSHCN)—defined as those requiring specialized services to manage chronic physical, developmental or mental health conditions (Ghandour et al. 2022)—widespread reductions in services contributed to discontinued therapies and adverse health outcomes (Allison and Levac 2022; Celona et al. 2023; DeSouza et al. 2024; Gigli and Graaf 2023; Merrick et al. 2023; Nicholas et al. 2022; Rast et al. 2025; Zhang et al. 2022), as well as greater social isolation and mental health problems compared with their peers (Dvorsky et al. 2023; Elgie et al. 2025; Geweniger et al. 2022; Samji et al. 2022; Summersett Williams et al. 2024).

Although the pandemic presented significant challenges, it also gave rise to unforeseen opportunities. The unprecedented explosion of virtual solutions made many areas of life more accessible, which can be especially helpful for those who manage complex healthcare needs, experience mobility issues or face accessibility barriers (Merrick et al. 2023; Pozniak et al. 2024; Zhang et al. 2022). For youth transitioning from paediatric to adult healthcare, virtual care provided opportunities to enhance autonomy by enabling independent management of appointments and more direct engagement in their care (Girdwood et al. 2023). Beyond improvements in service delivery, the pandemic also fostered space for strengthening family and social relationships, as well as a deeper engagement in hobbies (Bieniak et al. 2024; Nicholas et al. 2022).

The pandemic's ripple‐effects on health systems, education, employment and social connection continue to shape how young people live, learn and receive care. A deeper understanding of how the pandemic impacted YSHCN and their families can contribute enduring lessons to guide inclusive policies and practices for supporting YSHCN during periods of major transition and systemic disruption. As part of a larger patient‐oriented project on lessons learned from the experiences of YSHCN and their families during the COVID‐19 pandemic, the primary aim of this study was to *examine how, and to what extent, the COVID‐19 pandemic impacted the health and well‐being of YSHCN during their transition to adulthood*. A secondary, exploratory aim was to identify sources of heterogeneity in youth experiences with service delivery during the pandemic.

## 2 Methods

### 2.1 Setting and Study Design

We used a sequential, exploratory mixed methods design (Creswell and Plano Clark 2017) that included a qualitative study and a national survey. Data collection took place virtually, in Canada, between August 2022 and February 2024. Reporting of the study methods was guided by the Patient‐Centered Outcomes Research Institute's (PCORI) Methodology Standards for *Qualitative Methods and Mixed Methods Research* (Gaglio et al. 2020) and the Good Reporting of A Mixed Methods Study (GRAMMS) Checklist (O'Cathain et al. 2008; see Data S1). This study received ethical approval from the Hamilton Integrated Research Ethics Board (#14471) and the CHU Sainte‐Justine Research Ethics Board (2023‐4760), and all participants provided informed consent.

### 2.2 Study Population

The primary study population was YSHCN and their caregivers who were living in Canada. YSHCN included those between the ages of 16–28 years who have a developmental, physical or mental health condition.

### 2.3 Patient and Public Involvement

People with lived experience were actively involved throughout the research process. The project was initiated by members of the Canadian Transition Hub (Children's Healthcare Canada 2023), a national collaborative network that includes over 225 YSHCN, caregivers, researchers and clinicians across 10 Canadian provinces and territories. A young adult and two caregivers with lived experience were members of the research team. They regularly participated in project meetings, helped generate and refine research questions, provided feedback on study design and methods and co‐developed and piloted tools for data collection and analysis. They also supported recruitment, analysed data, interpreted findings through the lens of lived experience and contributed to dissemination. Ongoing feedback was also sought from the broader Canadian Transition Hub network.

### 2.4 Phase 1: Qualitative Study

#### 2.4.1 Research Question and Design

Phase 1 was guided by the following research question: *How has the COVID‐19 pandemic impacted healthcare, autonomy, relationships, vocation and lifestyle for YSHCN during their transition to adulthood?* This phase used an Interpretive Description approach (Thorne 2016), which is geared toward answering practical questions arising from real‐world concerns and is aimed at generating clinically relevant solutions. As a multidisciplinary team of health and social science researchers, clinicians and people with lived experience, an interpretive description approach allowed us to investigate the research question using a breadth of theoretical and practical knowledge.

#### 2.4.2 Sampling and Recruitment

Purposeful and snowball sampling methods were used to recruit YSHCN between the ages of 16–24 years or their caregiver proxies. Participants were recruited by self‐referral through advertising (e.g., social media, online newsletters) or through referrals from members of the Canadian Transition Hub. Interested YSHCN or their caregivers contacted the research team directly and were screened for eligibility. Purposeful sampling was used to recruit participants representing a range of experiences and contexts, including variation in geographical location, health status, age, gender and level of care complexity. Recruitment continued until the research team judged that sufficient depth and diversity of perspectives had been obtained to address the study objectives.

#### 2.4.3 Data Collection

The semi‐structured interview guide was designed to elicit stories from participants about how the COVID‐19 pandemic impacted their lives. Questions focused on the positive and negative experiences of YSHCN in five key areas: (i) healthcare; (ii) autonomy and independence; (iii) interpersonal relationships; (iv) education and employment; and (v) lifestyle and risk‐taking behaviours. These areas were selected as they reflect core developmental tasks of emerging adulthood and domains central to the transition to adult care (Arnett 2000; Betz et al. 2014; Nguyen et al. 2023). The interview guide (Data S2) was developed iteratively and was piloted with a youth–parent dyad on our research team.

Between August 2022 and April 2023, skilled interviewers conducted 21 semi‐structured interviews using the Zoom videoconferencing platform. All efforts were made to support YSHCN to participate in the interviews themselves, either independently or with the assistance of a support person or communication device. For those who were unable or required significant assistance to participate, a caregiver proxy responded on their behalf. All interviews were audio‐recorded, transcribed verbatim, then checked and de‐identified.

#### 2.4.4 Data Analysis

Interview data were analysed using rapid qualitative analysis, an action‐oriented approach that allows multidisciplinary teams and non‐specialists to efficiently identify themes in qualitative data (Hamilton and Finley 2019). This approach involved (1) extracting relevant concepts into a summary template for each participant (see Data S3), (2) charting the extracted data into a matrix and (3) performing matrix analyses to examine patterns and variations.

### 2.5 Phase 2: National Survey

#### 2.5.1 Research Question and Design

In Phase 2, we aimed to answer the following questions:

1. To what extent did the COVID‐19 pandemic create challenges and/or opportunities experienced by YSHCN and their families during the transition to adulthood? (primary quantitative question)
2. Which youth characteristics are associated with positive or negative impacts of pandemic‐related changes in service delivery? (secondary quantitative question)

In this phase, an anonymous cross‐sectional survey was administered in English or French to 516 YSHCN (or a caregiver proxy) between November and December 2023. The survey questions were informed by findings from Phase 1, a review of the literature and consultations with members of the Canadian Transition Hub.

#### 2.5.2 Sampling and Recruitment

Because the COVID‐19 pandemic was officially declared over in May 2023 (World Health Organization 2023)—prior to the start of Phase 2—we based Phase 2 eligibility criteria on youth participants' ages at the beginning of the pandemic in 2020 (CDC 2024). To support recruitment of a larger and more diverse sample, eligibility was expanded to YSHCN aged 17–28 years (or a proxy respondent), corresponding to ages 14–25 at the start of the pandemic. Recruitment for Phase 2 was conducted independently from Phase 1. We used Leger Opinion (LEO) Consumer Panel, an online consumer panel of 400 000 Canadians across 10 provinces and three territories, to recruit a target sample of 500 participants for the survey. To ensure that our sample was roughly representative of the Canadian study population, minimum quotas were set based on respondent type (90% self‐respondents, 10% proxy‐respondents), YSHCN age and gender, province of residence, and type and scope of healthcare needs. These quotas were confirmed using a series of screening questions. Participants received 1500 LEO points (equivalent to $1.50 CAD) for voluntary completion of the survey.

#### 2.5.3 Measures

For the present analysis, we examined the following measures from the survey (see Data S4 for relevant survey items).

*Youth demographics*
included age, gender identity, sexual orientation, race/ethnicity, province of residence and disability status.
*Healthcare needs*
were characterized by type (developmental, physical and/or mental) and scope of needs (number of healthcare services; medical complexity).
*Pandemic experiences*
were assessed by asking participants to endorse factors—derived from Phase 1 findings—that contributed to positive and negative experiences during the COVID‐19 pandemic. Positive factors included academic support, family support, relationships with friends and significant others, virtual services, financial assistance and positive coping strategies. Negative factors included lack of in‐person services, virtual services, difficulty maintaining relationships with friends, social media, negative news, social distancing and negative coping strategies.

#### 2.5.4 Statistical Analysis

Analyses were carried out in IBMSPSS software Version 27. All measures were summarized using descriptive statistics. To examine heterogeneity in experiences with service delivery, we first conducted exploratory bivariate analyses using chi‐square tests, followed by multivariable binary logistic regression. All variables were dichotomized, with the exception of gender identity, which was categorized into three groups. Three outcomes pertaining to pandemic‐related service delivery were analysed: positive impact of virtual services, negative impact of virtual services and negative impact of lack of in‐person services. Associations were examined between these three outcomes and the following youth characteristics: age group (17–22 vs. 23–28 years), gender identity (cisgender woman, cisgender man or gender diverse), type of healthcare needs (developmental, mental and/or physical) and level of service needs (< 3 vs. ≥ 3 services accessed regularly). Missing data in logistic regression analyses were handled using listwise deletion, with 41 cases (7.9%) excluded due to responses of ‘prefer not to answer’ or ‘I don't know’ for gender identity; no other variables had missing data. Multicollinearity was assessed using the variance inflation factor (VIF), with all VIF values below 5. A *p*‐value < 0.05 was considered statistically significant.

### 2.6 Mixed Methods Integration

Mixed methods integration occurred at two key stages of this study. First, insights from the qualitative phase informed the development of survey items, ensuring that survey questions reflected participants' real‐life experiences. Second, we integrated findings from both phases using joint displays, showcasing qualitative and quantitative results side‐by‐side to facilitate deeper exploration of results from each phase (McCrudden et al. 2021).

## 3 Results

### 3.1 Phase 1 Results (Qualitative)

In Phase 1, 21 interviews (17 English, 4 French) were conducted with 15 youth and 6 caregiver proxies from four Canadian provinces. Youth characteristics are reported in Table 1. Interviews explored youth and caregiver experiences during the pandemic, focusing on healthcare, independence, relationships, education and employment, and lifestyle.

**TABLE 1:** Youth characteristics for Phase 1 qualitative study (*N* = 21).

Characteristic	Mean (SD)	Frequency (%)
Age, years	19.1 (1.7)	
Gender identity^a^		
Cisgender woman		11 (52.4)
Cisgender man		8 (38.1)
Transgender/gender diverse^b^		2 (9.5)
Sexual orientation^a^		
Heterosexual		17 (81.0)
Two‐spirit/sexually diverse^c^		2 (9.5)
Prefer not to answer/unknown		2 (9.5)
Race/ethnicity^a^		
White		17 (81.0)
South Asian		2 (9.5)
Black		1 (4.8)
East Asian		1 (4.8)
West Asian/Arab		1 (4.8)
Province of residence		
Ontario		10 (47.8)
Québec		5 (23.8)
British Columbia		4 (19.0)
Alberta		2 (9.5)
Type of healthcare needs^a^		
Developmental		15 (71.4)
Physical		14 (66.7)
Mental		8 (38.1)

^a^ Participants could select multiple options.

^b^ Includes transgender woman, transgender man, non‐binary, two‐spirit, genderqueer, questioning and other term(s) not listed.

^c^ Includes lesbian, gay, bisexual, pansexual, two‐spirit, asexual and questioning.

#### 3.1.1 Healthcare

On the topic of healthcare, participants most often discussed the benefits and drawbacks of virtual care. Many appreciated the accessibility and convenience of virtual care options, particularly for routine visits or minor concerns. For example, Youth 11 explained:

Because of COVID we ended up doing some appointments over the phone and this was … pretty helpful for the endocrinology appointments because I've been going to her for so long that I didn't need to go to [City] to see her physically. Talking over the phone was sufficient enough.

However, others emphasized that the lack of in‐person services led to significant disruptions in care:

We stopped speech‐language therapy completely. We couldn't do it virtually. It was not possible because she's blind and deaf, and so we weren't able to do it. (Caregiver 20)

These mixed experiences highlight the dual impact of virtual care, facilitating access for some while leaving gaps for others who require more hands‐on support.

#### 3.1.2 Independence and Autonomy

Youth described varied experiences regarding independence. Some reported that the isolation imposed by the pandemic accelerated their self‐reliance:

It kind of made me independent over a shorter span of time than what I originally thought. (Youth 6)

In contrast, others experienced a loss of autonomy as concerns about infection risk in the face of underlying health conditions led them to rely more heavily on family members for their daily needs:

I couldn't get my own groceries because of being so immunocompromised … that was something I had to give to my parents to do for me, and I felt less independent, and that bothered me a lot because I worked so hard to work towards being an independent person, and it was like one step backwards. (Youth 2)

These experiences highlight how health‐related vulnerability and social restrictions shaped differing trajectories toward independence and adulthood.

#### 3.1.3 Relationships

Participants frequently reflected on how the pandemic reshaped their family and peer relationships. For some, increased time spent with family members fostered newfound closeness:

I have a younger brother … it was kind of my only socialization in those times. So, for sure it brought us a lot closer. (Youth 5)

For others, constant proximity led to heightened tension and conflict at home:

My sister and I were very irritable with each other. It was probably not doing so well for our relationship that we were constantly next to each other all day, every day, seven days a week. (Youth 17)

Friendships were also affected: Although some reported that support from friends and significant others helped alleviate feelings of isolation, the inability to meet in person was described as one of the most difficult aspects of the pandemic. Furthermore, YSHCN often engaged in greater COVID‐19 precautions than their peers, which sometimes led to social exclusion. Caregiver 19 observed how these differing views resulted in the fracturing of her daughter's friendships:

All her friendships changed because teens were not supportive. They were all gathering during COVID. She was not. And it's like they turned on her, like, ‘Oh, she's the one in the mask. She's the one who cares about COVID.’

Together, these accounts reflect how pandemic restrictions both strengthened and disrupted youths' social lives.

#### 3.1.4 Education and Employment

Disruption to education and career development was a recurring theme. Participants described delays in schooling, struggles with online learning, loss of structure provided by school and work, and disappointment over missed milestones:

Without school, without work, I really lost a structure that was necessary for my proper functioning. (Youth 5)

Class of 2020 got delayed two years and it was just a small ceremony in the high school auditorium. (Youth 10)

Youth also described positive impacts of the pandemic on their educational and work experiences. For example, some participants shared that the pandemic opened new employment opportunities, or that virtual learning afforded greater flexibility in the context of managing health needs:

I definitely had some work experiences that I've been able to find, and I don't know if I would have if COVID didn't exist. (Youth 6)

Part of the time when I was in Zoom school, I was in the hospital, so it was easy for me to catch up because I was already on Zoom. (Youth 12)

These experiences highlight how the pandemic created both barriers and unexpected opportunities for YSHCN, often leading to profound impacts on key developmental milestones.

#### 3.1.5 Lifestyle and Coping Behaviours

Youth and caregivers described the pandemic as a pervasive source of fear and stress, which was often exacerbated by negative news and social media. To cope, some youth adopted positive lifestyle strategies, such as engaging in physical activity and outdoor recreation:

I started going on lots of walks with my family in the pandemic… that kind of helped me stay active and obviously that's really good for my arthritis. (Youth 16)

Others, however, described maladaptive behaviours that negatively affected their well‐being, including changes in eating habits, social withdrawal and fear‐driven isolation:

Okay, so there were definitely unhealthy coping mechanisms… I relied on Uber Eats too much… it changed my diet a lot and that turned out unhealthy. (Youth 2)

I was very much just stuck in my house because I was too scared to leave it. (Youth 17)

These narratives reveal a complex interplay of vulnerability, resilience and adaptation across multiple domains.

### 3.2 Phase 2 Results (Quantitative)

#### 3.2.1 Youth Characteristics

In Phase 2, 516 respondents from 11 Canadian provinces and territories completed the survey, including 442 (85.7%) youth self‐respondents and 74 (14.3%) caregiver proxies. English responses comprised 77.5% (*n* = 400) of the returned surveys, with the remaining 22.5% (*n =* 116) completed in French. The majority identified mental healthcare needs (*n* = 359, 69.6%), followed by physical (*n* = 234, 45.3%) and developmental (*n* = 150, 29.1%) healthcare needs. A quarter (*n* = 134, 26.0%) reported higher service needs (≥ 3 services), and a small subset (*n* = 15, 2.9%) met criteria for medical complexity, as defined by Cohen et al. (2011) and the Canadian Institute for Health Information (2020). See Table 2 for full sample characteristics.

**TABLE 2:** Youth characteristics for Phase 2 national survey (*N* = 516).

Characteristic	Frequency (%)
Age group	
17–22 years (14–19 at start of pandemic)	203 (39.3)
23–28 years (20–25 at start of pandemic)	313 (60.7)
Gender identity^a^	
Cisgender woman	318 (61.6)
Cisgender man	100 (19.4)
Transgender/gender diverse^b^	66 (12.8)
Prefer not to answer/unknown	41 (7.9)
Sexual orientation^a^	
Heterosexual	343 (66.5)
Two‐spirit/sexually diverse^c^	195 (37.8)
Prefer not to answer/unknown	21 (4.1)
Race/ethnicity^a^	
White	394 (76.4)
South Asian	46 (8.9)
East Asian	27 (5.2)
Black	18 (3.5)
West Asian/Arab	18 (3.5)
Southeast Asian	14 (2.7)
Latin American	10 (1.9)
Other	19 (3.7)
First Nations, Métis or Inuk (Inuit)	40 (7.8)
Province of residence	
Ontario	200 (38.8)
Québec	127 (24.6)
Alberta	56 (10.9)
British Columbia	48 (9.3)
Manitoba	26 (5.0)
New Brunswick	22 (4.3)
Nova Scotia	13 (2.5)
Saskatchewan	12 (2.3)
Newfoundland and Labrador	9 (1.7)
Prince Edward Island	2 (0.4)
Yukon	1 (0.2)
Type of healthcare needs^a^	
Developmental	150 (29.1)
Physical	234 (45.3)
Mental	359 (69.6)
Scope of healthcare needs^a^	
Higher service needs (≥ 3 services)	134 (26.0)
Medically complex^d^	15 (2.9)
Identifies as a person with a disability	197 (38.2)

^a^ Participants could select multiple options.

^b^ Includes transgender woman, transgender man, non‐binary, two‐spirit, genderqueer, questioning and other term(s) not listed.

^c^ Includes lesbian, gay, bisexual, pansexual, two‐spirit, asexual and questioning.

^d^ Based on the definition of medical complexity used by the Canadian Institute for Health Information (CIHI 2020).

#### 3.2.2 Factors Contributing to Positive and Negative Pandemic Experiences

The survey built on the qualitative insights by examining which factors were most endorsed as contributing to positive and negative pandemic experiences. These factors are presented in a joint display (Table 3), alongside illustrative quotes from Phase 1 that informed their selection.

**TABLE 3:** Joint display of factors contributing to positive and negative experiences with illustrative quotes.

Factors contributing to positive experiences (*N* = 516)	Frequency (%)	Participant quotes from Phase 1
Family support	348 (67.4)	‘Probably the most positive thing was the time that we spent together as a family.’ (Caregiver 17)
Positive coping strategies (e.g., reading, going outside, physical activity)	310 (60.1)	‘My sister and I both play [piano] and we both really advanced our skills in it because it was something that we could focus on, especially when we were doing like school from home it was a good break from sitting at our desk all day.’ (Youth 11) ‘Daily exercise is a big one. And I also took up journaling during that time, which I still do.’ (Youth 6)
Relationships with friends	233 (45.2)	‘I kind of lived on Facetime with a lot of friends. My best friend and I would Facetime all day and just do our own thing.’ (Youth 2)
Virtual services	168 (32.6)	‘Those Zoom calls did make it easier to attend my appointments because I was starting university and it is hard right now, like my appointments are back in person and having to take the full day off to go get to [City] and then usually I have to take my mom with me… And so, the Zoom call I felt it was just a little bit more accessible.’ (Youth 16)
Relationships with significant other	156 (30.2)	‘I started to play some online games and that was where I met my husband … that was very positive for me, I think, in my life overall. And that was only because of the pandemic.’ (Youth 15)
Financial assistance	136 (26.4)	‘I took out $9000 in total from the [Canada Emergency Response Benefit] … for a period of time I was living heavily off of that.’ (Youth 15)
Academic support	110 (21.3)	‘Being online with school changed everything. I liked it because it meant I could stay in school and I was able to do a full course load which I've never actually been able to do with a learning disability in person.’ (Youth 2)
Other	13 (2.5)	

Factors contributing to negative experiences (*n* = 516)	Frequency (%)	Participant quotes from Phase 1
Lack of in‐person services	291 (56.4)	‘They did not get to see me in person. They did not get to see how sick I was in person.’ (Youth 2)
Negative news	289 (56.0)	‘I started religiously reading the news and trying to stay up to date on everything that was being released research‐wise on COVID. And so that probably in my head that was like, “Oh, if you know more about it, you are going to be less scared.” But honestly probably that was a little bit worse because the news kind of blew it out of proportion.’ (Youth 16)
Social distancing	264 (51.2)	‘I think there was a point where I feel like I did not hug someone in a really long time and that like physical connection was really sad.’ (Youth 2)
Not being able to keep relationships with friends	239 (46.3)	‘I did not get to see many friends during COVID. And I still have not seen many of my friends since COVID even started.’ (Youth 10)
Social media	185 (35.9)	‘[The pandemic] did allow people to be so exclusive because of the rules because they were like, “Oh, I'm having a party, but I can only have 20 people so I'm only going to invite these people.” And then they post it on social media, and I see it and I'm like, “Oh, that really … like I thought that person liked me, like oh, and they do not invite me.”’ (Youth 11)
Negative coping strategies (for example, alcohol or drug use)	150 (29.1)	‘During that time, I did struggle with things such as self‐harm and stuff just because it was so stressful and sometimes it got too much.’ (Youth 4)
Virtual services	128 (24.8)	‘She was also trying to learn online and that was a little too challenging for her. It's not like she's a regular child who could sit there and follow along with instructions, right? So, I had to be with her 24/7 during those times.’ (Caregiver 3)
Other	20 (3.9)	

##### 3.2.2.1 Positive Experiences

The most frequently endorsed factors contributing to positive experiences during the pandemic were family support (67.4%), positive coping strategies (60.1%) and relationships with friends (45.2%). These results emphasize the essential role of family and peer relationships in reducing isolation and providing stability during times of uncertainty. Positive coping strategies described in the interviews included physical activity, outdoor time and hobbies such as music or journaling. Perceptions on virtual services were mixed, with one‐third (32.6%) of survey respondents reporting a positive impact and one‐quarter (24.8%) reporting a negative impact. Other factors that contributed to positive experiences during the pandemic included relationships with significant others (30.2%), financial assistance (26.4%) and academic support (21.3%).

##### 3.2.2.2 Negative Experiences

The most frequently endorsed negative factors were lack of in‐person services (56.4%), exposure to negative news (56.0%) and social distancing (51.2%). Interview findings provide further insight through examples of youth being forced to discontinue therapies (e.g., physiotherapy or speech‐language therapy) due to the lack of in‐person options coupled with the difficulty of delivering these services online. Interview findings also expand on how news and social distancing contributed to heightened feelings anxiety and isolation. Other commonly cited negative influences included difficulties maintaining friendships (46.3%), social media (35.9%) and negative coping strategies (29.1%), such as self‐harm or substance use.

#### 3.2.3 Youth Characteristics Associated With Service Delivery Experiences

Chi‐square and logistic regression results examining associations between youth characteristics and experiences with pandemic‐related service delivery are presented in Table 4. The regression results indicate that youth with mental (aOR = 1.92, 95% CI 1.17–3.15) or developmental healthcare needs (aOR = 1.58, 95% CI 1.01–2.47) were more likely to endorse positive impacts of virtual services. Younger youth (aOR = 0.63, 95% CI 0.42–0.93) and those with mental healthcare needs (aOR = 1.88, 95% CI 1.18–2.98) were more likely to report negative impacts due to the lack of in‐person services. In bivariate analyses, cisgender women (*p* = 0.010) and those with developmental (*p* = 0.038) or higher service needs (*p* = 0.021) were also more likely to endorse negative impacts related to lack of in‐person services; however, these associations were not significant when adjusting for other variables. No youth characteristics were significantly associated with negative impacts of virtual services.

**TABLE 4:** Chi‐square and logistic regression results for associations between youth characteristics and pandemic‐related service delivery experiences.

Predictor	Virtual services contributed to positive experiences		Virtual services contributed to negative experiences		Lack of in‐person services contributed to negative experiences	
	Bivariate *p* ^a^	aOR (95% CI)^b^	Bivariate *p* ^a^	aOR (95% CI)^b^	Bivariate *p* ^a^	aOR (95% CI)^b^
Age group	0.431		0.732		0.014*	
17–22 years (14–19 at start of pandemic)		ref		ref		ref
23–28 years (20–25 at start of pandemic)		1.22 (0.81–1.82)		0.82 (0.53–1.27)		0.63 (0.42–0.92)*
Gender identity^c^	0.118		0.287		0.010*	
Cisgender woman		ref		ref		ref
Cisgender man		0.85 (0.47–1.53)		1.64 (0.80–3.36)		1.04 (0.58–1.87)
Gender diverse		0.64 (0.32–1.28)		2.13 (0.96–4.72)		0.66 (0.34–1.30)
Developmental healthcare needs	0.091	1.58 (1.01–2.47)*	0.786	1.13 (0.69–1.85)	0.038*	0.72 (0.47–1.12)
Mental healthcare needs	0.007*	1.92 (1.17–3.15)*	0.514	1.41 (0.83–2.40)	< 0.001*	1.88 (1.18–2.98)*
Physical healthcare needs	0.680	1.03 (0.66–1.59)	0.992	1.13 (0.70–1.83)	0.726	1.15 (0.74–1.77)
Service needs	0.349		0.773		0.021*	
< 3 services		ref		ref		ref
≥ 3 services		1.20 (0.76–1.88)		0.92 (0.56–1.52)		1.53 (0.97–2.41)

Abbreviations: aOR, adjusted odds ratio; CI, confidence interval; *p*, *p*‐value; ref, reference group.

^a^ *p*‐values from bivariate chi‐square tests.

^b^ Adjusted odds ratios and 95% confidence intervals from multivariable binary logistic regression.

^c^ Nine participants selected both cisgender woman and a gender‐diverse category and were categorized as gender diverse. Forty‐one cases had missing data, resulting in *N* = 475 for all regression analyses and chi‐square tests involving gender identity. *N* = 516 for all other chi‐square analyses.

* Significant findings, defined as *p* < 0.05.

## 4 Discussion

This patient‐oriented, sequential mixed methods study examined the impact of the COVID‐19 pandemic on Canadian YSHCN during their transition to adulthood. Although prior research has examined the pandemic's effect on YSHCN and on life transitions among the general population of emerging adults, limited attention has been paid to the intersection of these experiences. Our study addresses this gap, contributing a deeper and more nuanced understanding of the experiences unique to YSHCN as they navigated the pivotal transition to adulthood. We found that although many YSHCN experienced disruptions to healthcare, relationships and vocational development, others reported unexpected benefits such as increased autonomy, strengthened family bonds and improved accessibility through virtual platforms. These findings align with previous research highlighting the dual nature of the pandemic's impact on young people (Bieniak et al. 2024; Nicholas et al. 2022; Pozniak et al. 2024).

### 4.1 General Impacts of the Pandemic on YSHCN

The experiences of participants in this study reflect several global trends observed among youth with and without special healthcare needs during the pandemic. Consistent with previous reviews and population‐based studies (Dvorsky et al. 2023; Gigli and Graaf 2023; Merrick et al. 2023; Rast et al. 2025; Smyth and Nolan 2022), our study highlights the significant adverse effects of reduced access to health, education and community services for YSHCN. These disruptions were particularly pronounced for youth requiring in‐person care or specialized interventions, exposing inequities and systemic gaps in service delivery. Additionally, although social distancing and lockdown measures led to increased isolation, participants also reported strengthened relationships with family and friends—patterns that have previously been reported among the general youth population (Bieniak et al. 2024; Repo et al. 2023). Educational and vocational disruptions reported by participants in the current study likewise mirrored those described in other studies of emerging adults, including lost structure, delayed milestones and reduced opportunities for employment or training (Bieniak et al. 2024; Preetz et al. 2022; Smyth and Nolan 2022).

### 4.2 Nuanced Impacts on Life Transitions for YSHCN

Although YSHCN faced many similar challenges during the pandemic as the broader youth population, their greater reliance on health and social systems appeared to both intensify these shared challenges and contribute to experiences that were unique to this group. First, heightened health‐related vulnerability often prolonged and deepened social isolation, particularly for youth who remained more cautious than their peers as public health restrictions eased. Participants described feelings of exclusion and stigma from peers who dismissed precautions such as masking and social distancing. These findings align with those from a survey of Brazilian adolescents with chronic immunocompromised conditions (Lindoso et al. 2022), which found that fear of disease activity or complications was associated with psychosocial difficulties during the pandemic. Viewed through a life course lens (Halfon and Forrest 2018), these experiences—occurring during a developmental stage typically marked by identity and relationship formation—may have enduring effects on health and social trajectories well into adulthood.

Second, the pandemic affected youth autonomy in markedly different ways depending on individual circumstances and contexts. Although some participants were forced to become more self‐reliant due to social isolation, others—particularly those facing heightened health risks—relied more heavily on caregivers for safety and daily needs. Viewed through a relational autonomy lens, these divergent trajectories illustrate how autonomy is not an individual attribute, but rather, is shaped by a person's social, material and relational contexts (Gorter and Gibson 2016). From this perspective, increased reliance on others during the pandemic does not necessarily reflect diminished autonomy; rather, it underscores the inherent interdependence that characterizes everyday life for YSHCN.

Lastly, virtual and remote modalities had distinct implications for YSHCN. For some, online learning removed longstanding barriers by eliminating transportation challenges, reducing fatigue and enabling continuity of education during hospitalizations or recovery. Access to virtual healthcare can also create opportunities for greater autonomy by making it easier for youth to attend appointments independently—a finding consistent with prior work (Girdwood et al. 2023). However, these advantages were not universal. For example, our qualitative findings suggest that virtual modalities may be less effective or may deepen exclusion for those with developmental disabilities. Yet, our quantitative results found that negative impacts of virtual services were neither widely endorsed nor associated with any of the youth characteristics we examined. Rather, negative experiences with service delivery during the pandemic appeared to be largely driven by the absence or reduction of in‐person options, which was more likely to negatively impact younger youth and those with mental healthcare needs. Notably, youth with mental healthcare needs were more likely to experience both positive impacts of virtual services and negative impacts from lack of in‐person services, indicating the need for individualized approaches for this group. Together, our findings support that although virtual services may be beneficial for improving accessibility, they should complement rather than replace in‐person options. As such, this study reinforces existing calls for flexible approaches over one‐size‐fits‐all solutions (Nicholas et al. 2022; Pozniak et al. 2024).

### 4.3 Implications for Practice and Policy

The COVID‐19 pandemic catalysed innovations with the potential to advance accessibility and inclusion. However, many of these adaptations also deepened inequities and social exclusion. For example, some caregiver participants reported that their young person was unable to engage through virtual formats, leading to inequitable access to health care and exclusion from learning. Others shared that underlying health vulnerabilities contributed to greater social isolation or alienation from peers due to diverging views on public health measures. These insights draw attention to the pervasive ableism embedded in everyday environments and made evident in pandemic responses (Schormans et al. 2021). Our findings further highlight the variability in experiences—both within and across groups of YSHCN—underscoring the need for flexible, inclusive and resilient health, education and social systems that are capable of adapting to future disruptions while safeguarding vulnerable and historically marginalized groups (Nicholas et al. 2022; Schormans et al. 2021). To promote inclusive healthcare, clinicians should offer flexible options—backed by policy—that balance the accessibility of virtual care with the indispensable value of in‐person services, tailored to individual needs and circumstances. For policymakers, lessons from the pandemic emphasize the importance of embedding accessibility and inclusion across public systems and developing national disability strategies to protect vulnerable populations (de Kraus Camargo 2024; United Nations 2020). Furthermore, our study contributes to growing evidence on the pandemic's broad impacts on youth, reinforcing the urgent need for greater investments in youth vocational development, financial stability and mental health. Lastly, achieving these goals requires meaningful engagement with youth, caregivers and people with special healthcare needs/disabilities in policy and programme design, ensuring that those most affected by systemic gaps are central to future crisis preparedness and recovery planning.

### 4.4 Strengths and Limitations

Strengths of this study include its patient‐oriented design, pan‐Canadian scope and sequential mixed methods approach. People with lived experience were engaged throughout the research process, enhancing the relevance, accessibility and credibility of the study. Flexible participation options (e.g., independent, caregiver‐supported or proxy) and the use of complementary methods were intentionally employed to support inclusion of diverse perspectives, particularly those of youth with developmental disabilities or complex healthcare needs who are often excluded from research. The qualitative phase enabled in‐depth exploration of these underrepresented perspectives, whereas the subsequent survey captured patterns across a broader population of YSHCN. Although this complementarity is a strength of the mixed methods design, it also resulted in differences in sample composition across phases. As such, integrated findings should be interpreted as shared patterns across related but not identical subgroups.

Limitations related to survey design, recruitment and sample composition should be considered when interpreting these findings. First, the cross‐sectional nature of the survey—though appropriate for an exploratory study—precludes causal interpretation. Second, despite using minimum sampling quotas based on national demographics, the sample was not fully representative of the Canadian YSHCN population (e.g., women were overrepresented). Third, recruitment through the LEO panel resulted in a sample of individuals who may differ from other YSHCN in important ways (e.g., greater engagement with health or social systems, comfort with technology or stronger motivation to share their experiences), and perspectives of YSHCN who face barriers to participating in online research may be underrepresented. Fourth, although proxy reporting enabled the participation of youth with significant communication barriers, it may also introduce bias and may not accurately represent youth perspectives. Lastly, as the inferential analyses were exploratory, potential interaction effects between variables were not examined and may warrant further investigation.

## 5 Conclusions

This study provides new insight into how the COVID‐19 pandemic broadly affected the lives of YSHCN during the transition to adulthood, not only in relation to health but also across interconnected areas including independence, relationships, education and employment, and daily lifestyle. The pandemic disrupted access to healthcare, interrupted key developmental milestones and exacerbated pre‐existing inequities. At the same time, it catalysed virtual adaptations that improved accessibility for some and highlighted the importance of family support and adaptive coping strategies as protective factors. Lessons learned from the experiences of YSHCN should inform the development of more flexible, inclusive systems that sustain recent accessibility gains and strengthen support for YSHCN through post‐pandemic recovery and future disruptions.

## Author Contributions

**Lin Li:** conceptualization, formal analysis, funding acquisition, methodology, visualization, writing – original, writing – review and editing. **Jan Willem Gorter:** conceptualization, data curation, formal analysis, funding acquisition, investigation, methodology, project administration, resources, supervision, validation, writing – review and editing. **Karen Cook:** conceptualization, data curation, formal analysis, funding acquisition, investigation, methodology, project administration, validation, visualization, writing – original, writing – review and editing. **Alene Toulany:** conceptualization, validation, writing – review and editing. **Paula Robeson:** methodology, resources, writing – review and editing. **Jessica Geboers:** conceptualization, data curation, methodology, visualization, writing – review and editing. **Dilshad Kassam‐Lallani:** conceptualization, data curation, methodology, validation, writing – original, writing – review and editing. **Danijela Grahovac:** conceptualization, funding acquisition, investigation, resources, software, validation, writing – review and editing. **Aline Bogossian:** formal analysis, methodology, writing – review and editing. **Rocio Gutierrez Rojas:** data curation, investigation, resources, writing – review and editing. **Barb Galuppi:** data curation, formal analysis, investigation, methodology, project administration, resources, supervision, validation, visualization, writing – original, writing – review and editing. **Dayle Mccauley:** conceptualization, data curation, formal analysis, funding acquisition, methodology, project administration, writing – review and editing. **Anne Fournier:** conceptualization, funding acquisition, investigation, methodology, project administration, resources, supervision, validation, visualization, writing – review and editing.

## Funding

This work was funded by the Canadian Institutes of Health Research (UIP 178856).

## Ethics Statement

This study received ethical approval from the Hamilton Integrated Research Ethics Board (#14471) and the CHU Sainte‐Justine Research Ethics Board (2023‐4760).

## Consent

All participants provided informed consent.

## Conflicts of Interest

The authors declare no conflicts of interest.

## Supporting information


**Data S1:** Supporting information.

**Data S2:** Supporting information.

**Data S3:** Supporting information.

**Data S4:** Supporting information.

## Data Availability

The data that support the findings of this study are available upon request from the corresponding author. These data are not publicly available due to privacy or ethical restrictions.

## References

[cch70294-bib-0001] Allison, K. M. , and D. E. Levac . 2022. “Impact of the COVID‐19 Pandemic on Therapy Service Delivery and Functioning for School‐Aged Children With Disabilities in the United States.” Disability and Health Journal 15, no. 2: 101266. 10.1016/j.dhjo.2021.101266. 35115260 PMC8730530

[cch70294-bib-0002] Arnett, J. J. 2000. “Emerging Adulthood. A Theory of Development From the Late Teens Through the Twenties.” American Psychologist 55, no. 5: 469–480. 10842426

[cch70294-bib-0003] Betz, C. L. , M. E. Ferris , J. F. Woodward , M. J. Okumura , S. Jan , and D. L. Wood . 2014. “The Health Care Transition Research Consortium Health Care Transition Model: A Framework for Research and Practice.” Journal of Pediatric Rehabilitation Medicine 7, no. 1: 3–15. 10.3233/PRM-140277. 24919934

[cch70294-bib-0004] Bieniak, K. H. , H. Bedree , N. Geanous , et al. 2024. “Thematic Analysis of COVID‐19's Impacts on Transitions Among Emerging Adults.” Health Care Transitions 2: 100052. 10.1016/j.hctj.2024.100052. 39712585 PMC11657545

[cch70294-bib-0005] Canadian Institute for Health Information . 2020. “Children and Youth With Medical Complexity in Canada.” CIHI. https://www.cihi.ca/en/children‐and‐youth‐with‐medical‐complexity‐in‐canada.

[cch70294-bib-0006] CDC . 2024. “CDC Museum COVID‐19 Timeline.” Centers for Disease Control and Prevention. https://www.cdc.gov/museum/timeline/covid19.html.

[cch70294-bib-0007] Celona, C. A. , K. Jackman , and A. Smaldone . 2023. “Emergency Department Use by Young Adults With Chronic Illness Before and During the COVID‐19 Pandemic.” Journal of Emergency Nursing 49, no. 5: 755–764. 10.1016/j.jen.2023.04.006. 37256242 PMC10133889

[cch70294-bib-0008] Children's Healthcare Canada . 2023. “Transition to Adult Care Hub.” https://www.childrenshealthcarecanada.ca/en/networks‐and‐hubs/transition‐to‐adult‐care‐hub.aspx.

[cch70294-bib-0009] Cohen, E. , D. Z. Kuo , R. Agrawal , et al. 2011. “Children With Medical Complexity: An Emerging Population for Clinical and Research Initiatives.” Pediatrics 127, no. 3: 529–538. 10.1542/peds.2010-0910. 21339266 PMC3387912

[cch70294-bib-0010] Creswell, J. W. , and V. L. Plano Clark . 2017. Designing and Conducting Mixed Methods Research. 3rd ed. SAGE Publications.

[cch70294-bib-0011] de Kraus Camargo, O. 2024. “Why Canada Needs a National Disability Strategy.” The Conversation. 10.64628/AAM.tmq4vhrgj.

[cch70294-bib-0012] DeSouza, A. , D. Wang , J. J. Wong , et al. 2024. “Prevalence of Unmet Rehabilitation Needs Among Canadians Living With Long‐Term Conditions or Disabilities During the First Wave of the COVID‐19 Pandemic.” Archives of Physical Medicine and Rehabilitation 105, no. 2: 268–279. 10.1016/j.apmr.2023.07.010. 37541355

[cch70294-bib-0013] Dvorsky, M. R. , D. Shroff , W. B. Larkin Bonds , A. Steinberg , R. Breaux , and S. P. Becker . 2023. “Impacts of COVID‐19 on the School Experience of Children and Adolescents With Special Educational Needs and Disabilities.” Current Opinion in Psychology 52: 101635. 10.1016/j.copsyc.2023.101635. 37451025 PMC10275652

[cch70294-bib-0014] Elgie, M. , C. K. Y. Chan , C. Bedard , and M. A. Ferro . 2025. “Changes in Psychological Distress in Children With Physical Illness and Their Parents During the COVID‐19 Pandemic.” Child: Care, Health and Development 51, no. 3: e70082. 10.1111/cch.70082. 40387373 PMC12087426

[cch70294-bib-0015] Gaglio, B. , M. Henton , A. Barbeau , et al. 2020. “Methodological Standards for Qualitative and Mixed Methods Patient Centered Outcomes Research.” BMJ 371: m4435. 10.1136/bmj.m4435. 33361103 PMC7756351

[cch70294-bib-0016] Geweniger, A. , M. Barth , A. D. Haddad , et al. 2022. “Impact of the COVID‐19 Pandemic on Mental Health Outcomes of Healthy Children, Children With Special Health Care Needs and Their Caregivers—Results of a Cross‐Sectional Study.” Frontiers in Pediatrics 10: 759066. 10.3389/fped.2022.759066. 35223688 PMC8866820

[cch70294-bib-0017] Ghandour, R. M. , A. H. Hirai , and M. K. Kenney . 2022. “Children and Youth With Special Health Care Needs: A Profile.” Pediatrics 149, no. Supplement 7: e2021056150D. 10.1542/peds.2021-056150D. 35642877

[cch70294-bib-0018] Gigli, K. H. , and G. Graaf . 2023. “Changes in Use and Access to Care for Children and Youth With Special Health Care Needs During the COVID‐19 Pandemic.” Journal of Pediatric Health Care Special Issue: The Effects of the COVID‐19 Pandemic for Children and Youth With Special Health Care Needs 37, no. 2: 185–192. 10.1016/j.pedhc.2022.09.008. 36216644 PMC9489986

[cch70294-bib-0019] Girdwood, T. C. , J. L. Goralski , M. E. Ferris , et al. 2023. “Perceptions of Caregivers and Adolescents/Young Adults With Cystic Fibrosis Regarding Health Care Transition Readiness During the COVID‐19 Pandemic: A Qualitative Study.” Health Care Transitions 1: 100011. 10.1016/j.hctj.2023.100011. 39712998 PMC11657345

[cch70294-bib-0020] Gorter, J. , and B. Gibson . 2016. “Independence in Adulthood: Ethical Challenges in Providing Transition Services for Young People With Neurodevelopmental Impairments.” In Ethics in Child Health: Principles and Cases in Neurodisability, edited by P. Rosenbaum , G. Ronen , B. Dan , J. Johannesen , and E. Racine , 259–266. Mac Keith Press.

[cch70294-bib-0021] Halfon, N. , and C. B. Forrest . 2018. “The Emerging Theoretical Framework of Life Course Health Development.” In Handbook of Life Course Health Development, edited by N. Halfon , C. B. Forrest , R. M. Lerner , and E. M. Faustman , 19–43. Springer International Publishing. 10.1007/978-3-319-47143-3_2. 31314301

[cch70294-bib-0022] Hamilton, A. B. , and E. P. Finley . 2019. “Qualitative Methods in Implementation Research: An Introduction.” Psychiatry Research 280: 112516. 10.1016/j.psychres.2019.112516. 31437661 PMC7023962

[cch70294-bib-0023] Juvonen, J. , L. M. Lessard , N. G. Kline , and S. Graham . 2022. “Young Adult Adaptability to the Social Challenges of the COVID‐19 Pandemic: The Protective Role of Friendships.” Journal of Youth and Adolescence 51, no. 3: 585–597. 10.1007/s10964-022-01573-w. 35103932 PMC8805132

[cch70294-bib-0024] Lindoso, L. , C. Astley , L. B. Queiroz , et al. 2022. “Physical and Mental Health Impacts During COVID‐19 Quarantine in Adolescents With Preexisting Chronic Immunocompromised Conditions.” Jornal de Pediatria 98, no. 4: 350–361. 10.1016/j.jped.2021.09.002. 34699770 PMC8506207

[cch70294-bib-0025] Mazzolani, B. C. , F. I. Smaira , C. Astley , et al. 2021. “Changes in Eating Habits and Sedentary Behavior During the COVID‐19 Pandemic in Adolescents With Chronic Conditions.” Frontiers in Pediatrics 9: 714120. 10.3389/fped.2021.714120. 34966698 PMC8711628

[cch70294-bib-0026] McCrudden, M. T. , G. Marchand , and P. A. Schutz . 2021. “Joint Displays for Mixed Methods Research in Psychology.” Methods in Psychology 5: 100067. 10.1016/j.metip.2021.100067.

[cch70294-bib-0027] Merrick, H. , H. Driver , C. Main , et al. 2023. “Impacts of Health Care Service Changes Implemented due to COVID‐19 on Children and Young People With Long‐Term Disability: A Mapping Review.” Developmental Medicine and Child Neurology 65, no. 7: 885–899. 10.1111/dmcn.15503. 36649197

[cch70294-bib-0028] Nguyen, L. , C. Dawe‐McCord , M. Frost , et al. 2023. “A Commentary on the Healthcare Transition Policy Landscape for Youth With Disabilities or Chronic Health Conditions, the Need for an Inclusive and Equitable Approach, and Recommendations for Change in Canada.” Frontiers in Rehabilitation Sciences 4: 1305084. https://www.frontiersin.org/articles/10.3389/fresc.2023.1305084. 38192636 10.3389/fresc.2023.1305084 PMC10773791

[cch70294-bib-0029] Nicholas, D. B. , W. Mitchell , J. Ciesielski , A. Khan , and L. Lach . 2022. “A Qualitative Examination of the Impact of the COVID‐19 Pandemic on Individuals With Neuro‐Developmental Disabilities and Their Families.” Journal of Child and Family Studies 31, no. 8: 2202–2214. 10.1007/s10826-022-02336-8. 35855733 PMC9281199

[cch70294-bib-0030] O'Cathain, A. , E. Murphy , and J. Nicholl . 2008. “The Quality of Mixed Methods Studies in Health Services Research.” Journal of Health Services Research & Policy 13, no. 2: 92–98. 10.1258/jhsrp.2007.007074. 18416914

[cch70294-bib-0031] Pozniak, K. , A. Swain , G. Currie , et al. 2024. “What Supports and Services Post COVID‐19 Do Children With Disabilities and Their Parents Need and Want, Now and Into the Future?” Frontiers in Public Health 12: 1294340. 10.3389/fpubh.2024.1294340. 38655511 PMC11036871

[cch70294-bib-0032] Preetz, R. , J. Greifenberg , J. Hülsemann , and A. Filser . 2022. “Moving Back to the Parental Home in Times of COVID‐19: Consequences for Students' Life Satisfaction.” International Journal of Environmental Research and Public Health 19, no. 17: 10659. 10.3390/ijerph191710659. 36078374 PMC9518347

[cch70294-bib-0033] Rast, J. E. , K. H. Koffer Miller , J. Bromberg , J. Ventimiglia , K. A. Anderson , and L. L. Shea . 2025. “Changes in Child Health Care, Health, and Caregiver Mental Health During the COVID‐19 Pandemic in Children With Autism and Special Health Care Needs.” Maternal and Child Health Journal 29, no. 1: 78–86. 10.1007/s10995-024-04020-3. 39576377 PMC11805851

[cch70294-bib-0034] Repo, J. , S. Herkama , T. Yanagida , and C. Salmivalli . 2023. “Transition to Emerging Adulthood During the COVID‐19 Pandemic: Changes in Anxiety and the Role of Inclusion/Exclusion Experiences.” European Journal of Developmental Psychology 20, no. 4: 649–665. 10.1080/17405629.2022.2122434.

[cch70294-bib-0035] Samji, H. , J. Wu , A. Ladak , et al. 2022. “Review: Mental Health Impacts of the COVID‐19 Pandemic on Children and Youth—A Systematic Review.” Child and Adolescent Mental Health 27, no. 2: 173–189. 10.1111/camh.12501. 34455683 PMC8653204

[cch70294-bib-0036] Schormans, A. F. , S. Hutton , M. Blake , K. Earle , and K. J. Head . 2021. “Social Isolation Continued: Covid‐19 Shines a Light on What Self‐Advocates Know Too Well.” Qualitative Social Work 20, no. 1–2: 83–89. 10.1177/1473325020981755. 34253956 PMC8261336

[cch70294-bib-0037] Sharma, A. K. , K. Jain , K. Mulchandani , et al. 2024. “Navigating the Challenges: Exploring the Association Between COVID‐19 Lockdowns and Eating Behavior in University Students: A Systematic Review and Investigation of Factors Impacting Ed Levels.” Hormone Molecular Biology and Clinical Investigation 45: 85–98. 10.1515/hmbci-2023-0049. 39138656

[cch70294-bib-0038] Smyth, E. , and A. Nolan . 2022. “Disrupted Transitions? Young Adults and the COVID‐19 Pandemic.” [Report]. ESRI. 10.26504/rs142.

[cch70294-bib-0039] Summersett Williams, F. , I. Zaniletti , A. R. Masonbrink , et al. 2024. “Substance Use Emergency Department Visits Among Youths With Chronic Conditions During COVID‐19.” JAMA Network Open 7, no. 10: e2435059. 10.1001/jamanetworkopen.2024.35059. 39365582 PMC11581538

[cch70294-bib-0040] Thorne, S. 2016. Interpretive Description. Routledge. 10.4324/9781315426259.

[cch70294-bib-0041] United Nations . 2020. “Pandemic Reveals How Excluded Are Society's Most Marginalized, Secretary‐General Says, Launching Policy Brief on Persons With Disabilities and COVID‐19.” https://press.un.org/en/2020/sgsm20074.doc.htm.

[cch70294-bib-0042] World Health Organization . 2023. “WHO Director‐General's Opening Remarks at the Media Briefing—5 May 2023.” https://www.who.int/director‐general/speeches/detail/who‐director‐general‐s‐opening‐remarks‐at‐the‐media‐briefing‐‐‐5‐may‐2023.

[cch70294-bib-0043] Zhang, S. , Y. Hao , Y. Feng , and N. Y. Lee . 2022. “COVID‐19 Pandemic Impacts on Children With Developmental Disabilities: Service Disruption, Transition to Telehealth, and Child Wellbeing.” International Journal of Environmental Research and Public Health 19, no. 6: 3259. 10.3390/ijerph19063259. 35328947 PMC8951004

