# Supplementary material for: Growing Up Through a Pandemic: A Mixed‐Methods Study of How the COVID‐19 Pandemic Shaped the Transition to Adulthood for Youth With Special Healthcare Needs and Their Families
Source: Child Care Health Dev. 2026 May 10;52:e70294. doi: 10.1111/cch.70294 (PMC13158332; doi:10.1111/cch.70294)
Supplement: Supplementary file 1 — Data S1: Supporting information. [file CCH-52-e70294-s001.docx]

**Supplemental Table 1.** Good Reporting of A Mixed Methods Study (GRAMMS) Checklist. From: O’Cathain, A., Murphy, E., & Nicholl, J. (2008). The Quality of Mixed Methods Studies in Health Services Research. *Journal of Health Services Research & Policy*, *13*(2), 92–98. <https://doi.org/10.1258/jhsrp.2007.007074>

| **Guideline** | **Section & Page** |
| --- | --- |
| Describe the justification for using a mixed methods approach to the research question | Methods: Setting and Study Design |
| Describe the design in terms of the purpose, priority and sequence of methods | Methods: Setting and Study Design |
| Describe each method in terms of sampling, data collection and analysis | Methods: Phase 1: Qualitative Study & Phase 2: National Survey |
| Describe where integration has occurred, how it has occurred and who has participated in it | Methods: Mixed Methods Integration |
| Describe any limitation of one method associated with the present of the other method | Discussion: Strengths and Limitations |
| Describe any insights gained from mixing or integrating methods | Discussion: Strengths and Limitations;  Results: Phase 2: Quantitative and Mixed Methods Results;  Table 3: Joint Display of Factors Contributing to Positive and Negative Experiences with Illustrative Quotes |
| **Note:** Due to space limitations in the main manuscript, further details about each item in this table are described in Supplemental Table 2 below. | |

**Supplemental Table 2.** Methodological standards for qualitative and mixed methods patient centered outcomes research. From: Gaglio, B., Henton, M., Barbeau, A., Evans, E., Hickam, D., Newhouse, R., & Zickmund, S. (2020). Methodological standards for qualitative and mixed methods patient centered outcomes research. *BMJ*, *371*, m4435. <https://doi.org/10.1136/bmj.m4435>

| **Standards for qualitative methods** | |
| --- | --- |
| *QM-1: State the qualitative approach to research inquiry, design, and conduct* | |
| A. Identify and describe evidence gaps that support the need for a qualitative component(s) of the study | Rationale provided in the background section. Due to limited research on how the pandemic impacted youth with special health care needs (YSHCN) during their transition to adulthood, an exploratory study was warranted. The exploratory nature of the research question lends itself well to a qualitative approach, which can provide in-depth and nuanced insights on the phenomenon. A qualitative approach also allows for the exploration of emergent themes that may otherwise be missed when using quantitative methods alone. |
| B. Identify the qualitative approach that will be used, including the purpose, why it is an appropriate approach to answer the research question(s), and how it will be operationalized | **Approach**: Interpretive description (ID)  **Purpose**: To explore how the COVID-19 pandemic impacted the lives of YSHCN in the context of their transition to adulthood.  **Rationale for approach:** ID applies a disciplinary lens to address complex experiential questions in health research. As a multidisciplinary team of health researchers, clinicians, and people with lived experience, an ID approach allowed us to bring a breadth of theoretical and practical knowledge to the research process. |
| C. Describe the types of data to be collected, strategies for data collection, and when the data will be collected | Data were collected via semi-structured interviews using Zoom (alternative methods were offered for youth who required adapted communication methods) between August 2022 and April 2023. |
| D. Describe how confidentiality will be maintained through data collection, management, analysis, and reporting | Interview transcripts were de-identified after data collection, and prior to analysis. Only de-identified data were used for analysis and reporting. |
| E. State the computer software program used to assist with analysis | Templated summaries were created in Microsoft Word/Excel. |
| *QM-2: Select and justify appropriate qualitative methods sampling strategy* | |
| A. Describe and provide the rationale for the sampling strategy, including how the strategy flows logically from the qualitative approach and how it fits the research question(s) | Purposeful sampling was used to obtain a breadth of narrative data from individuals with various lived contexts. This strategy allowed the voices of those who are less represented in large samples to be heard, such as youth with medical complexity. |
| B. Explain the anticipated sample size, detail any variation in sampling that may occur over the course of study, and state the criteria for deciding when no further sampling is necessary | We anticipated conducting 30-40 interviews. We did not aim to achieve information saturation, but rather to collect a broad spectrum of experiences to inform the development of the survey in phase 2. We stopped after 21 interviews to stay within our study timeline and supplemented our interview findings with a literature search and reflections on the interpretations from Transition Hub members, including youth, caregivers, researchers, and clinicians. |
| C. Describe how the methods will ensure that the data capture the depth of experiences of the participants or phenomenon of interest | A subgroup of our research team—including young adult and parent partners—collaborated to develop the interview guide. The development process was iterative, and led to modifications to optimize inclusion of those who required support to participate.  We trained 4 interviewers (2 each for English and French interviews). One of our parent partners and her son who is a YSHCN participated in a pilot interview using a communication device as a training exercise for interviewers.  Participants could use words or pictures to talk about their experiences and questions were provided in advance to allow the participant to prepare their answers. |
| *QM-3: Link the qualitative data analysis, interpretations, and conclusions to the study question* | |
| A. State who will be involved in the data analysis and interpretation and describe how their qualifications, training, and expertise equip them to understand and address the complexities and challenges unique to qualitative methods | Six research team members were involved in analysis, including experienced researchers, trainees, and research staff with experience in qualitative research (BLINDED). |
| B. Describe data analysis procedures and their link to the study’s research questions | Rapid qualitative analysis was used to synthesize the data collected from interviews. This action-oriented approach involves using a template to extract relevant concepts from individual transcripts and then using matrix analyses to map variation across respondents.  Summary templates were developed collaboratively by two research staff (BLINDED) and refined by the qualitative subgroup (BLINDED). The template was piloted prior to use to confirm suitability for extracting and synthesizing data to answer the study’s research questions. |
| C. Describe the process by which inferences and themes will be identified and developed as well as how this process is congruent with the chosen qualitative approach and its methodology | Following the pilot analysis, each transcript was analyzed by one research team member (BLINDED), who created a templated summary. The templated summaries were charted into a matrix and analyzed by the research team to identify key concepts that would inform the development of the subsequent survey. This approach was compatible with ID, as it was accessible to a multidisciplinary team of researchers and lay people, allowing the data to be interpreted through multiple disciplinary lenses. |
| D. Describe how conclusions will be derived and how they relate to interpretations and content of the original data | Findings from the analysis were shared with the whole research team to draw conclusions and inform the development of the survey. We also leveraged the expertise of the Transition Hub to provide input on the qualitative interpretations and survey development. |
| *QM-4: Establish trustworthiness* and credibility of qualitative research* | |
| A. State how documentation regarding all phases of the analysis will be captured. Multiple data collection methods (e.g., interviews, focus groups, observations) and/or experts with diverse backgrounds can be used to increase trustworthiness, in addition to an inter-coder reliability process | All interviews were audio-recorded, transcribed verbatim, then checked for accuracy and de-identified by the same interviewer. Detailed notes and memos were recorded to document the study process and key study decisions. Regular meetings were held with the research team and minutes were documented.  In addition to interview data, we supplemented our qualitative findings with a literature review and input from the Transition Hub members. The research team was multidisciplinary, including people with lived experience (youth/caregiver), researchers, and clinicians, contributing to the trustworthiness of the findings (see below). |
| B. To enhance credibility, discuss three distinct elements: rigorous techniques and methods, the role of the qualitative researcher, and the value of participants’ perspectives and experiences. Credibility must be explained and demonstrated in the analysis in at least one of the following three ways: reflexivity, negative case analysis, and/or member checking | Rigorous techniques have been described above pertaining to data collection and analysis (e.g., iterative processes and piloting of data collection and analysis tools), findings interpretation, and study documentation. Through an ID approach, we actively sought to leverage the diverse backgrounds of the research team in shaping and interpreting the research (role of the researcher). Through purposeful sampling and in-depth interviews, we sought to include the experiences and perspectives of a diverse group of participants.  **Reflexivity:** The qualitative findings reflect the diverse perspectives of our research team, including lived experience experts, researchers, and clinicians. Clinical disciplines represented in our team include medicine (developmental pediatrics, adolescent medicine, child and adolescent psychiatry), social work, occupational therapy, nursing, and psychology. Specific areas of research expertise included: health and social care transitions for youth in various contexts (e.g., with medical complexity, raised in the foster system, or with intellectual disabilities), ethical/social issues and social participation of people with disabilities and chronic health conditions, and community engaged research. Lived experience experts included a mother of an adolescent with GMFCS Level V Cerebral Palsy, a journalist and young adult with Cerebral Palsy, and a nurse practitioner and parent of a youth with a chronic health condition.  **Member checking:** The interviewer provided a summary at the end of the interview to allow the participant to confirm, change, or clarify any aspects of their stories.  **Negative case analysis:** We asked about both positive and negative experiences to explore varied experiences and possible deviations from expected patterns. |
| **Standards for mixed methods research** | |
| *MM-1: Specify how mixed methods are integrated across design, data sources, and/or data collection phases* | |
| A. State which mixed methods approach will be used and describe how it will inform the study procedures | Sequential, exploratory mixed methods (QUAL 🡪 QUAN). First, the QUAL phase explored *how* the COVID-19 pandemic has impacted YSHCN during their transition to adulthood. Next, the QUAN phase investigated *to what extent* these impacts were experienced among a larger sample that is representative of the population. As the research aims were exploratory in nature, the benefits of both qualitative and quantitative methods were leveraged to more comprehensively understand the impacts of the COVID-19 pandemic on YSHCN. |
| B. Describe whether the quantitative and qualitative methods will be sequential, concurrent, or a mixture of both, over time | Sequential (QUAL 🡪 QUAN) |
| *MM-2: Select and justify appropriate mixed methods sampling strategy* | |
| A. Provide a clear description of the relationship between the sampling techniques and the generation of different types of data | **QUAL:** sampling techniques aimed to obtain a breadth of perspectives.  **QUAN:** sampling techniques aimed to improve generalizability by obtaining a sample that representative of the Canadian population of YSHCN. |
| B. Describe the sampling strategies and outline the temporality with which they will take place as they relate to selected qualitative and quantitative methodologies, including a justification of the emergence of other samples that may arise during the study, as applicable | **QUAL:** purposeful and snowball sampling strategies were used to obtain a variety of experiences to inform survey development. Recruitment occurred via social media and the Transition Hub network. QUAL sampling preceded QUAN sampling.  **QUAN:** The sample was drawn from an online consumer panel of 400,000 Canadians. Quotas were used to ensure that our sample was roughly representative of the Canadian study population. Quotas were set based on respondent type, YSHCN age and gender, province of residence, and type and scope of health care needs. |
| *MM-3: Integrate data analysis, data interpretation, and conclusions* | |
| A. Describe the analytic approaches to integration and demonstrate how the analysis plan is congruent with the study design and aims, and that it has been developed based on the methodological approach | Rapid qualitative analysis informed the methods and materials for the subsequent QUAN phase. The emergent design makes sense for an exploratory study, where there is limited knowledge of the phenomenon. |
| B. Identify the order of study components and the points of integration. State who will conduct the integration; describe how their qualifications, training, and expertise equip them to understand and address the complexities and challenges unique to mixed methods analysis; and state how integrated analyses will proceed in terms of the qualitative and quantitative components | Points of integration:  1) Between the QUAL and QUAN phases: qualitative findings informed quantitative survey;  2) Following the analysis of both phases: qualitative and quantitative findings were integrated using joint displays.  Integration was conducted by research team members with expertise in qualitative and mixed methods research (i.e., qualitative co-investigators and research staff). |
| C. Describe the approach used to interpret integrated data and how conclusions are supported by the context of original qualitative and quantitative findings. Address divergent findings from both qualitative and quantitative components, as well as method-specific biases across the methods | In addition to the research team, integrated findings were shared through consultations with the Transition Hub, where youth, parents, and providers shared their interpretations and thoughts on the qualitative, quantitative, and integrated data. Their feedback was considered in the process of deciding which results should be prioritized and brought forward from each phase. Our research and writing team also included people with lived experience, who have provided feedback on the interpretations and conclusions.  Method-specific biases reflect the different demographics of each phase. For example, because the survey collected data from a large sample representative of the Canadian population, underrepresented groups (such as youth with medical complexity or developmental disabilities) would have less overall impact on the findings. The qualitative methods helped to address this issue, as we made sure to capture the perspectives of people from these underrepresented groups. |
