# Supplementary material for: Growing Up Through a Pandemic: A Mixed‐Methods Study of How the COVID‐19 Pandemic Shaped the Transition to Adulthood for Youth With Special Healthcare Needs and Their Families
Source: Child Care Health Dev. 2026 May 10;52:e70294. doi: 10.1111/cch.70294 (PMC13158332; doi:10.1111/cch.70294)
Supplement: Supplementary file 2 — Data S2: Supporting information. [file CCH-52-e70294-s002.docx]

**Interview Guide**

*[Instructions to Interviewer: The interviewer should allow the participant to participate in any form that allows the youth to provide information. Establish how to best communicate (e.g., if there are nonverbal cues for the interviewer to be aware of) For individuals with literacy or visual impairments, information will be read to the participant. For hearing impairments, we will allow the individual to either bring an interpreter, provide response via augmentative/alternative communication or via email or can put the questions up on the screen to be read by the participant (these are examples and not an exhaustive list). For those with severe impairments who may not be able to respond to the questions, the interview should be modified to allow for yes/no or multiple-choice questions or we will allow for a proxy responder.]*

**Introduction:**

Hi ________________, my name is _____________ and I am an [role] from [institution]. Thank you for setting aside the time today to speak with me. We are conducting a research study to explore the impact of the COVID-19 pandemic on Youth with Special Health Care Needs during their transition to adult services (between 16-24 years of age).

First, I’d like to define what I mean by transition to adulthood and transfer to adult services as they are two distinct (or different) concepts. When I use the word ‘transition’ I am referring to the time in life when youth start to move into more ‘adult’ roles and responsibilities; a time when youth might take on new tasks, achieve new milestones, or have new or different goals and aspirations. I also understand that every person and family will have different ideas, values, and beliefs about what it means to be an adult. So, I am interested in hearing from you.

When I use the word ‘transfer’ in our interview, I will be referring to the time that you moved from child focused to new or different services for adults. These can be health, education, and or social services.

So, while transition is a broad topic that touches all aspects of a person’s life, transfer is focused on services.

In this interview, we will explore some of your experiences with transition and transfer, and what it was like for you to go through different challenges and opportunities during the COVID-19 pandemic. I would like to explore topics around autonomy (or independence), education, employment, relationships, and health or risk-taking behaviours.

During the interview, if there is a question you don’t want to answer, just say, “pass.” If you are curious about why I am asking a certain question or questions, I invite you to ask me and I will do my best to explain. We can also pause or stop the interview at any time, and you can withdraw from the study at any time if you wish. You will not face any negative consequences and I will not be upset if you choose to withdraw. Our conversations today will be confidential and we will do everything we can to protect you and your family’s identities. When we report this information, we will remove anything that may identify you or your family (e.g., names, city or school information, employer names). Participation in other studies does not impact on your ability to participate in this study.

It is important for us to record the interview so that we have an accurate and complete record of our discussion to analyze at a later date. Without the audio recording, we will not be able to use information from the interview in our study and you will not be able to participate. Are you ok with me recording our conversation?

**Demographic Questions:**

1. How old are you?

1. What gender identity/ies do you identify with?
   1. Female
   2. Male
   3. Transgender Male
   4. Transgender Female
   5. Non-Binary
   6. Two-Spirit
   7. Genderqueer
   8. Questioning
   9. Term(s) not listed here: _______________
   10. Prefer not to state
2. What sexual orientation(s) do you identify with?
   1. Straight
   2. Lesbian
   3. Gay
   4. Bisexual
   5. Pansexual
   6. Two-Spirit
   7. Asexual
   8. Questioning
   9. Term(s) not listed here: _______________
   10. Prefer not to state
3. What group(s) do you identify as?
4. White
5. South Asian (e.g., East Indian, Pakistani, Sri Lankan, etc.)
6. Chinese
7. Black
8. Filipino
9. Latin American
10. Arab
11. Southeast Asian (e.g., Vietnamese, Cambodian, Laotian, Thai, etc.)
12. West Asian (e.g., Iranian, Afghan, etc.)
13. Pacific Islander
14. Indigenous
15. Metis
16. Term(s) not listed here: _______________
17. Prefer not to state
18. What country were you born in?
19. What is your status in Canada?
    1. Canadian Citizen
    2. Permanent Resident
    3. Other:
20. Can you provide me with the first three digits of your postal code?

**Interview Questions**

As described earlier, we are interested in exploring the impact of the COVID-19 pandemic on Youth with Special Health Care Needs. We would like to know in what ways the pandemic has impacted your health during your transition from pediatric to adult services. We would like to hear about both positive and negative changes to your life or experiences in your education, employment, and health that you feel is attributed to the COVID-19 pandemic.

1. Please tell me about your disability or special healthcare need. Can you describe it to me?

PROBES:

Level of complexity of the participant’s special healthcare need: What is your health condition? Because of this condition, do you need support to do things in your everyday life? Do you take medication? Do you require any special treatments? Do you use adaptive devices? What type of help or support do you have or need but don’t have? Are there things that you have started managing on your own (e.g., making appointments, going places on your own, asking for support to do new things that you want to do). Stage of transition: Have you recently moved from one service to another, for example, changing a doctor, changing to a new service centre, school etc.?

As described earlier, we are interested in knowing the ways in which the COVID-19 pandemic has impacted you during your transition from pediatric to adult services. We would like to hear both positive and negative changes to your life (such as changes related to education, employment, or health) that you feel is attributed to the COVID-19 pandemic.

1. Let’s start with an example or story of a positive or negative change that impacted you (in the area of your independence, relationships, education, employment, or health management) and that you feel is attributed to the COVID-19 pandemic. [Other option: show a picture and describe what is meaningful about it with respect to experiences during the COVID-19 pandemic]

*[Based on youth’s story, start at the appropriate section below]*

**Healthcare Self-Management (services you receive, your ability to express yourself and your rights)**

How would you say that the COVID-19 pandemic has impacted the healthcare services you received?

PROBES:

Frequency: Did it impact how often you saw your healthcare providers?

Access: Did it impact your access to healthcare providers, in other words, your ability to go to see or speak to a healthcare provider (s) when you needed to?

Mode/Type of Visit: Did you visit your healthcare providers in-person? Online? Mix of both? What were the benefits/drawbacks of the visits you had during the pandemic?

**Autonomy/Independence:**

Becoming an adult is often connected to increasing one's independence (e.g., trying new things, taking on more responsibility, making choices and decisions for your own life). How would you say that the COVID-19 pandemic has had an impact on your ability to be more independent?

PROBES:

Living situation: Where you live and with whom

Social life/Community involvement: Seeing or spending time with friends and participating in activities that you like

Personal support worker: Receiving support from a caregiver/personal support worker. Were they available when you needed them?

Accessible transportation/mobility aids: Were you able to arrange transportation, get new or repair existing mobility aids as you needed?

If the during the pandemic you felt more/less independent, how do you think this impacted your health (physical, mental, functional)?

**Relationships**

*Family*

How would you say that the COVID-19 pandemic has impacted your relationships with your friends and family?

PROBES:

Spend more/less time together, need more/less help, feeling connected/disconnected

**Education/Employment**

How would you say that the COVID-19 pandemic has impacted your ability to participate in school or work?

PROBES:

Transportation, Accessibility, Public health measures (e.g., masking requirements), decisions regarding attending school/gaining or continuing employment

**Lifestyle**

Did you experience increased stress related to the COVID-19 pandemic in your everyday life and if so, what were those stressors? How did you cope? What worked to help you feel less stressed? Did you add or take away activities to feel better?

PROBES:

Exercise and diet; Increased alcohol, drug use or vaping/smoking, healthy sexuality, safety to self or others (e.g., suicidal ideation)

Did you make changes to your exercise routine? Why and how?

Did you make changes to your diet, or did you change the things you were eating? Why and how?

Did you change how much alcohol you drank? (more or less use)

Did you change your smoking or vaping habits?

Have you started using drugs or made changes in substance use since the beginning of the pandemic?

Have there been positive or negative changes in your sex life?

Have you felt that you might want to harm yourself or others?

**After the Interview:**

I would just like to sum up some of the major points that I have heard.

*[Interviewer provide brief summary].*

Did I understand these key takeaways correctly (in terms of the challenges and opportunities)? Is there anything important that I missed or that you want to add? What were the key contributing factors to the positive or negative experience? What are the lessons learned? What can we take away from your experience to improve things in the future?

Thank you for taking the time to speak with me and share your experiences today.
