# Supplementary material for: Growing Up Through a Pandemic: A Mixed‐Methods Study of How the COVID‐19 Pandemic Shaped the Transition to Adulthood for Youth With Special Healthcare Needs and Their Families
Source: Child Care Health Dev. 2026 May 10;52:e70294. doi: 10.1111/cch.70294 (PMC13158332; doi:10.1111/cch.70294)
Supplement: Supplementary file 3 — Data S3: Supporting information. [file CCH-52-e70294-s004.docx]

**Interview Summary Template**

**Notes:**

- This summary is not interpretative, it is a sketch of what the participant talked about in that interview, to enhance our accessibility to what is in that data. Condense data to its most powerful/impact statement.
- Add quotes within table sparingly. Power quotes – words that capture the “essence” of the interview, e.g., potential to be journal title.
- Use Analytic memo column to highlight points for discussion, for recommendations, observation of patterns across interviews (alignment or points of convergence), interpretations, reflections.
- Each Data Summary cell should have an entry. Write “Not discussed” if the interviewer did not ask/participant did not discuss the topic.
- This is the consistent template we will use for the rapid analysis of each transcript. If you identify a “new” domain – please add it to the end of the chart under “Other”.

| **Domain** | **Data Summary** | **Quotes** | **Analytic Memos** | **Suggested Survey Question(s)** |
| --- | --- | --- | --- | --- |
| 1. Special healthcare need |  |  |  |  |
| 1. Adaptive devices/ Supports in everyday life |  |  |  |  |
| 1. Medication/treatments/specialists |  |  |  |  |
| 1. Stage of transition (health care transition, other significant transitions) |  |  |  |  |
| 1. Positive impact of COVID-19 pandemic on healthcare |  |  |  |  |
| 1. Negative impact of COVID-19 pandemic on healthcare |  |  |  |  |
| 1. Positive impact of COVID-19 pandemic on autonomy/independence |  |  |  |  |
| 1. Negative impact of COVID-19 pandemic on autonomy/independence |  |  |  |  |
| 1. Positive impact of COVID-19 pandemic on relationships with family |  |  |  |  |
| 1. Negative impact of COVID-19 pandemic on relationships with family |  |  |  |  |
| 1. Positive impact of COVID-19 pandemic on relationships with friends |  |  |  |  |
| 1. Negative impact of COVID-19 pandemic on relationships with friends |  |  |  |  |
| 1. Positive impact of COVID-19 pandemic on romantic relationships |  |  |  |  |
| 1. Negative impact of COVID-19 pandemic on romantic relationships |  |  |  |  |
| 1. Positive impact of COVID-19 pandemic on education |  |  |  |  |
| 1. Negative impact of COVID-19 pandemic on education |  |  |  |  |
| 1. Positive impact of COVID-19 pandemic on employment |  |  |  |  |
| 1. Negative impact of COVID-19 pandemic on employment |  |  |  |  |
| 1. Positive impact of COVID-19 pandemic on lifestyle (habits/activities/routines) |  |  |  |  |
| 1. Negative impact of COVID-19 pandemic on lifestyle (habits/activities/routines) |  |  |  |  |
| 1. Positive coping strategies |  |  |  |  |
| 1. Negative coping strategies |  |  |  |  |
| 1. Overall key contributing factors to positive experiences |  |  |  |  |
| 1. Overall key contributing factors to negative experiences |  |  |  |  |
| 1. Key takeaways to improve things in the future |  |  |  |  |
| 1. Impact of COVID-19 pandemic on physical or mental health |  |  |  |  |
| 1. OTHER  - Impact of COVID-19 pandemic on caregiver |  |  |  |  |
