# Supplementary material for: Growing Up Through a Pandemic: A Mixed‐Methods Study of How the COVID‐19 Pandemic Shaped the Transition to Adulthood for Youth With Special Healthcare Needs and Their Families
Source: Child Care Health Dev. 2026 May 10;52:e70294. doi: 10.1111/cch.70294 (PMC13158332; doi:10.1111/cch.70294)
Supplement: Supplementary file 4 — Data S4: Supporting information. [file CCH-52-e70294-s003.docx]

The following survey questions were used in the present analysis.

# SCREENING QUESTIONS:

1. **Where in Canada do you live?**
2. British Columbia
3. Alberta
4. Saskatchewan
5. Manitoba
6. Ontario
7. Quebec
8. New Brunswick
9. Nova Scotia
10. Prince Edward Island
11. Newfoundland
12. Northwest Territories
13. Yukon
14. Nunavut
15. **What is your age?** _____ years

**S3. Do you have any of these special healthcare need(s)?**

Some conditions may fall into multiple categories. Please select all that apply.

1. **Developmental healthcare needs**

This may include difficulty learning, remembering, or concentrating, Down syndrome, autism, mental impairment due to lack of oxygen at birth, or other conditions that affect how the brain develops.

1. **Mental healthcare needs**

This may include emotional, psychological, or mental health conditions such as anxiety, depression, bipolar disorder, substance use disorder, eating disorder, etc.

1. **Physical healthcare needs**

This may include health problems or long-term conditions involving the structure or function of body systems such as heart, brain, lungs, kidneys, etc. This may also include difficulty seeing even when wearing glasses or contact lenses, difficulty hearing even when using a hearing aid or cochlear implant, difficulty walking, using stairs, using your hands or fingers, or doing other physical activities, etc. I do not have a child between the ages of 17-28 years with special healthcare need(s)

1. No special healthcare need(s)

**S4. What gender identity/ies do you identify with?**

Please select all that apply.

1. Cisgender Woman
2. Cisgender Man
3. Transgender Woman
4. Transgender Man
5. Non-Binary
6. Two-Spirit
7. Genderqueer
8. Questioning
9. Prefer not to answer

**S6.** How many different healthcare services, providers or teams do you access regularly for your special healthcare need(s)? **If you see a therapist or specialist on their own, count that as 1 healthcare service. If you see a team of people together at the same appointment, also count that as 1 service**. ______

**S7. Is your special healthcare need(s) medically complex?**

When we talk about people with medical complexity, we mean they have all the following:

- complex chronic condition(s) affecting more than one body systems or affecting a single body system severely enough to require specialty care (e.g., cardiology) and sometimes hospitalization, **and**
- limitations in performing usual activities because of their healthcare needs (and in some cases, requiring the assistance of medical technologies), **and**
- high health care utilization (for example, requiring hospitalization or needing regular care from multiple healthcare providers or teams), **and**
- high family-identified needs that have a significant impact on the family/caregiver.

1. Yes
2. No

**..................................................................................................................................................**

# QUESTIONS ABOUT YOU (SELF-COMPLETE VERSION):

**SC1. Do you identify as a person with a disability?**

1. Yes
2. No

**SC3. What sexual orientation(s) do you identify with?**

Please select all that apply.

1. Heterosexual
2. Lesbian
3. Gay
4. Bisexual
5. Pansexual
6. Two-Spirit
7. Asexual
8. Questioning
9. Term(s) not listed here
10. Prefer not to state

**SC4. Are you First Nations, Métis, or Inuk (Inuit)?**

1. No
2. Yes, First Nations
3. Yes, Métis
4. Yes, Inuk (Inuit)

**SC5. What group(s) do you identify as?**

Please select all that apply.

1. White
2. South Asian (e.g., East Indian, Pakistani, Sri Lankan)
3. Chinese
4. Black
5. Filipino
6. Arab
7. Latin American
8. Southeast Asian (e.g., Vietnamese, Cambodian, Laotian, Thai)
9. West Asian (e.g., Iranian, Afghan)
10. Korean
11. Japanese
12. Other

# QUESTIONS ABOUT YOUR COVID-19 PANDEMIC EXPERIENCE

**SC20. What factors do you think led to *positive experiences* during the COVID-19 pandemic?**

1. Academic support
2. Family support
3. Relationships with friends
4. Relationships with significant other
5. Virtual services
6. Financial assistance
7. Positive coping strategies (for example, reading, going outside, physical activity)
8. Other

**SC21. What factors do you think led to *negative experiences* during the COVID-19 pandemic?**

- 1. Lack of in-person services
  2. Virtual services
  3. Not being able to keep relationships with friends
  4. Social media
  5. Negative news
  6. Social distancing
  7. Negative coping strategies (for example, alcohol or drug use)
  8. Other
